# Accelerometer informed time-energy budgets reveal the importance of temperature to the activity of a wild, arid zone canid

**DOI:** 10.1186/s40462-021-00246-w

**Published:** 2021-03-18

**Authors:** Jack Tatler, Shannon E. Currie, Phillip Cassey, Anne K. Scharf, David A. Roshier, Thomas A. A. Prowse

**Affiliations:** 1 Invasion Science & Wildlife Ecology Lab, University of Adelaide, Adelaide, SA 5005 Australia; 2 Department of Evolutionary Ecology, Leibniz Institute for Zoo and Wildlife Research, Alfred-Kowalke Str. 17, 10315 Berlin, Germany; 3 Department of Migration, Max Planck Institute of Animal Behavior, Am Obstberg 1, 78315 Radolfzell, Germany; 4 Australian Wildlife Conservancy, PO Box 8070, Subiaco East, WA 6008 Australia; 5 School of Mathematical Sciences, University of Adelaide, Adelaide, SA 5005 Australia

**Keywords:** Behaviour, Dingo, Energy expenditure, ODBA, Temperature, Time-energy budget

## Abstract

**Background:**

Globally, arid regions are expanding and becoming hotter and drier with climate change. For medium and large bodied endotherms in the arid zone, the necessity to dissipate heat drives a range of adaptations, from behaviour to anatomy and physiology. Understanding how apex predators negotiate these landscapes and how they balance their energy is important as it may have broad impacts on ecosystem function.

**Methods:**

We used tri-axial accelerometry (ACC) and GPS data collected from free-ranging dingoes in central Australia to investigate their activity-specific energetics, and activity patterns through time and space. We classified dingo activity into stationary, walking, and running behaviours, and estimated daily energy expenditure via activity-specific time-energy budgets developed using energy expenditure data derived from the literature. We tested whether dingoes behaviourally thermoregulate by modelling ODBA as a function of ambient temperature during the day and night. We used traditional distance measurements (GPS) as well as fine-scale activity (ODBA) data to assess their daily movement patterns.

**Results:**

We retrieved ACC and GPS data from seven dingoes. Their mass-specific daily energy expenditure was significantly lower in summer (288 kJ kg^− 1^ day^− 1^) than winter (495 kJ kg^− 1^ day^− 1^; *p* = 0.03). Overall, dingoes were much less active during summer where 91% of their day was spent stationary in contrast to just 46% during winter. There was a sharp decrease in ODBA with increasing ambient temperature during the day (*R*^2^ = 0.59), whereas ODBA increased with increasing T_a_ at night (*R*^2^ = 0.39). Distance and ODBA were positively correlated (*R* = 0.65) and produced similar crepuscular patterns of activity.

**Conclusion:**

Our results indicate that ambient temperature may drive the behaviour of dingoes. Seasonal differences of daily energy expenditure in free-ranging eutherian mammals have been found in several species, though this was the first time it has been observed in a wild canid. We conclude that the negative relationship between dingo activity (ODBA) and ambient temperature during the day implies that high heat gain from solar radiation may be a factor limiting diurnal dingo activity in an arid environment.

**Supplementary Information:**

The online version contains supplementary material available at 10.1186/s40462-021-00246-w.

## Introduction

Movement is the primary contributor to active energy expenditure in most vertebrates [1–3]. Animals move to improve their individual fitness through, for example, access to food resources, to avoid predators, or to find mates. Underlying these behaviours is the need to balance energy acquisition and expenditure, which ultimately determines an animal’s behaviour and location in the landscape [4–6]. In addition, variation in the landscape structure such as substrate, vegetation type, and elevation will have varying movement costs [7, 8]. Given that animals tend not to position themselves randomly [5, 9–11], understanding purposive movements and use of space provides insight into the ecophysiology of mobile taxa.

The presence of medium and large carnivores in a landscape can strongly influence the structure and function of ecosystems [12–14]. In fact, mammalian predators are often used as bio-indicators in the event of human-induced ecosystem disruption given their local extinction can trigger a trophic cascade [15]. Quantifying the behaviour and resulting energy demands of free-ranging carnivores is therefore useful for predicting their resource requirements and subsequent selection of patchily distributed resources across the landscape.

Australia’s largest terrestrial predator, the dingo *Canis dingo*, is a medium-sized eutherian carnivore that persists in a wide range of environments [16]. Dingoes are a highly mobile species that traverse large areas to acquire resources, and maintain social ties and territorial boundaries. As a result, the decision to move is biologically significant and likely to vary at fine (e.g., daily) and broad (e.g., seasonal) temporal scales, as well as spatially. For populations in the harsh, resource-limited deserts of central Australia where risks of hyperthermia are high, survival depends on making choices that minimise behavioural energetic expenditure (and evaporative water loss), whilst optimising the acquisition of food, shelter, and water resources needed for survival [17].

How wild animals balance their energetics through time and space is increasingly being studied by integrating movement data with activity-specific time-energy budgets [18, 19]. Animal movement can be reliably captured by animal-attached accelerometers (ACC) that measure changes in acceleration in up to three axes [20]. As energy expenditure is a function of activity, behaviours can then be linked to activity specific measures of energy expenditure to produce robust time-energy budgets in free-ranging animals. Time-energy budgets, the categorisation of energy cost per activity integrated over the time spent performing that activity, provide a reliable estimate of daily energy expenditure [21]. Measuring energetic costs for free-ranging and highly mobile predators like dingoes is challenging and, to date, we have a limited understanding of how physiological capacities and environmental variables affect their movement and use of space.

It has been suggested that an individual’s maximal energy expenditure may be constrained by their ability to dissipate heat [22] and therefore bodily processes that generate heat (e.g., movement, digestion etc.) trade-off within a total limit defined by heat dissipation capacity. For large (> 10 kg) mammals in arid regions, the ability to lose heat is limited by low surface area to volume ratios and thus cooling can be slow [23]. This is increasingly important to understand in the wake of global climate change as arid regions are likely to experience even hotter temperatures and prolonged droughts [24]. As a first response, individuals are most likely to adjust their short term behaviour before longer term physiological adaptations or range adjustments [23].

Investigating how dingoes behave in an already challenging environment could potentially provide us insights into how other arid zone predators cope under climate change. Here, we used ACC and GPS data collected from free-ranging dingoes in central Australia to investigate their behaviour-specific energetics and activity patterns through time and space, and uncover the trade-offs imposed by their arid habitat. We classified broad classes of behaviour from ACC data and used it to estimate daily energy expenditure via activity-specific time-energy budgets. We explored the dingo’s behaviour at different times of the day and year, and examined daily patterns of activity in response to ambient temperature as an indication of behavioural thermoregulation. We expected that dingoes would be most active at night and that during periods of high temperature dingoes would be inactive to reduce the risk of hyperthermia. Finally, we explored how dingoes behave and partition their energy in relation to landscape features.

## Methods

### Study area and species

**Fig. 1:**
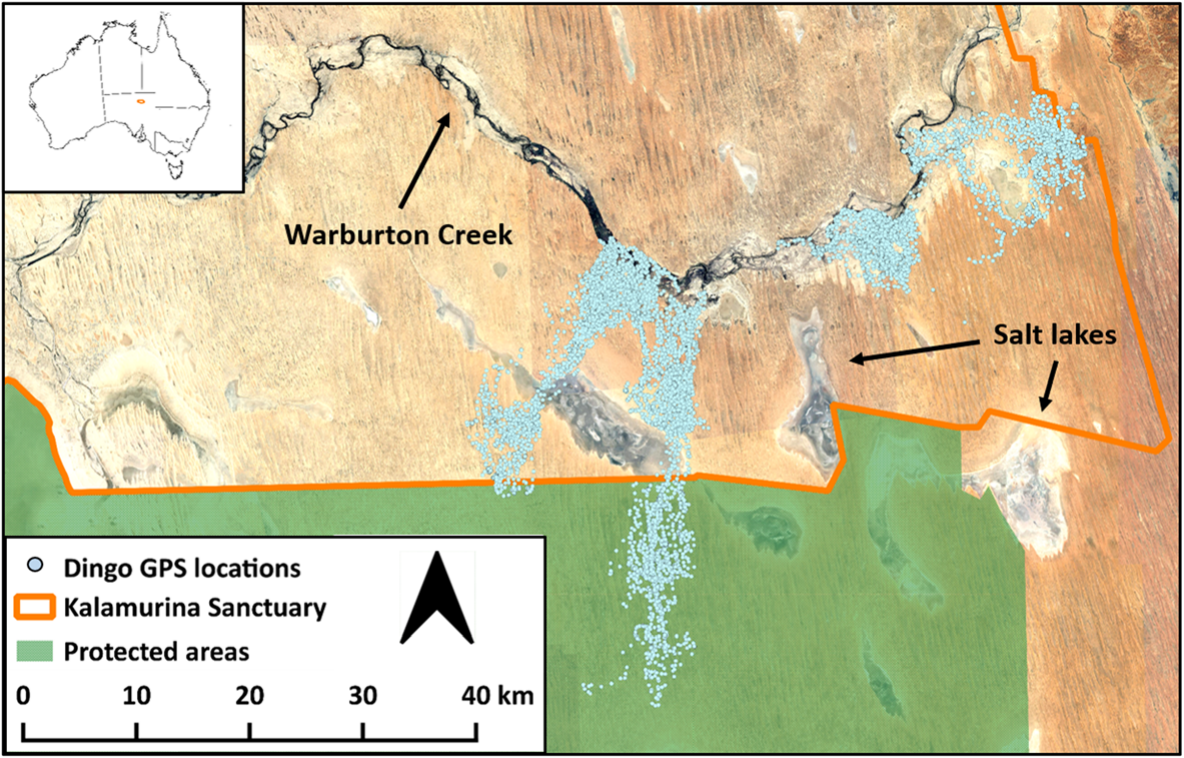
Tracking data from seven dingoes at Kalamurina Sanctuary. Inset displays the location of Kalamurina in central Australia. The Warburton Creek is the only major watercourse on the eastern side of the study site, and it is bordered by shrubland and desert woodland along its length. The majority of Kalamurina consists of sand dunes and flats

Our study took place from April 2016 to May 2018 at Kalamurina Wildlife Sanctuary (hereafter ‘Kalamurina’), a 6670 km^2^ conservation area owned and managed by Australian Wildlife Conservancy, and located at the intersection of three of Australia’s central deserts: the Simpson, Tirari, and Sturt’s Stony Desert (27°48’S, 137°40’E, UTM Zone 54S; Fig. 1). The site adjoins protected areas to the north and south to create a 64,064 km^2^ contiguous area that is managed for conservation. The region’s climate is arid with a median annual rainfall of 133.5 mm, and it is characterised by very hot summers and mild winters; mean daily temperatures ranging from 23 °C – 38 °C in the hottest month and 6 °C – 20 °C in the coldest month (with mid-afternoon being the hottest part of the day) [25]. It is located in the Simpson-Strzelecki Dunes Bioregion and the dominant landform is sand dunes (< 18 m), with scattered floodplains, claypans, and salt lakes. The dune swales are characterised by chenopod shrubland where the main vegetation are species of *Acacia*, *Eremophila*, and *Atriplex*. Extensive coolabah *Eucalyptus coolabah* woodlands exist along the banks and floodplains of the larger watercourses.

The dingoes at Kalamurina possess high levels of dingo ancestry [26], making this study the first assessment of energetics in a wild population of pure dingoes. European rabbits *Oryctolagus cuniculus*, c. 1.6 kg comprise the bulk of their diet, but they also consume reptiles, birds, invertebrates, and vegetation [27].

### Data collection, cleaning, and processing

Dingoes were captured using Victor Soft Catch® #3 leg-hold traps modified with Paws-I-Trip pans and a Jake Chain Rig (Professional Trapp Supplies, Molendinar, Queensland). These traps and modifications are designed to reduce the impact on the trapped limb [28]. All traps were set within close proximity (< 20 m) to tracks and checked twice daily within 3 h of sunrise and sunset. All methodology employed as part of this study were ethically reviewed and approved (University of Adelaide Animal Ethics Committee S-2015-177A). We fit 19 dingoes with ACC-GPS collars (Telemetry Solutions, Concord, CA, USA) that were equipped with tri-axial accelerometers (LISD2H, ST Microelectronics, USA) programmed to sample changes in acceleration at 1 Hz (one sample per second) and orientated so that the x, y, and z-axes recorded acceleration along the sway, heave, and surge planes, respectively. To increase the temporal window of data collection, accelerometers were scheduled to record on a one-day on, three-days off sampling regime. We programmed the GPS to record a location every 15 min on the days the ACC was active. Six collars were recovered via triggering the drop-off mechanism, and one was recovered by re-trapping.

To limit the effect of abnormal behaviour that might occur as a result of capture and collaring, we discarded any GPS and ACC data recorded during the 24 h immediately following release. Data were also discarded if they had a horizontal dilution of precision ≥9 (a measure of GPS accuracy) or occurred after the collar had dropped-off. We were able to retrieve ACC and GPS data from seven dingoes. Three individuals were tracked during winter 2016 and four during summer 2017–2018. All data manipulation and analyses were conducted in the R software environment for statistical and graphical computing (version 3.5.1 [29];).

### Dingo behaviour, ODBA, and energetic expenditure

We classified wild dingo behaviours from the ACC data using the Random Forest model described in Tatler et al. [30]. This supervised-learning approach required ACC data to be manually classified into behaviours, which was achieved by observing captive dingoes (*n* = 3) that were equipped with tri-axial ACC units programmed to sample at 1 Hz. The predictive performance of the model was assessed using out-of-sample validation and resulted in the accurate classification of 14 dingo behaviours. However, for the purposes of this paper, we were only interested in general movement patterns that would influence daily energy expenditure. Therefore, we trained a new Random Forest model (with the same set of parameters as in *30*) to identify five classes of movement: lying down, sitting, standing, walking, and running (Table 1). Grouping the raw ACC data from the highest and lowest intensity behaviours increased the sample size and improved the accuracy at which our model classified these movements (Table 1). Once we had classified our wild dingo ACC data into five behaviours, we relabelled Lying, Standing, and Sitting behaviours to ‘Stationary’ as these behaviours are so similar that they are unlikely to differ energetically.

**Table 1:**
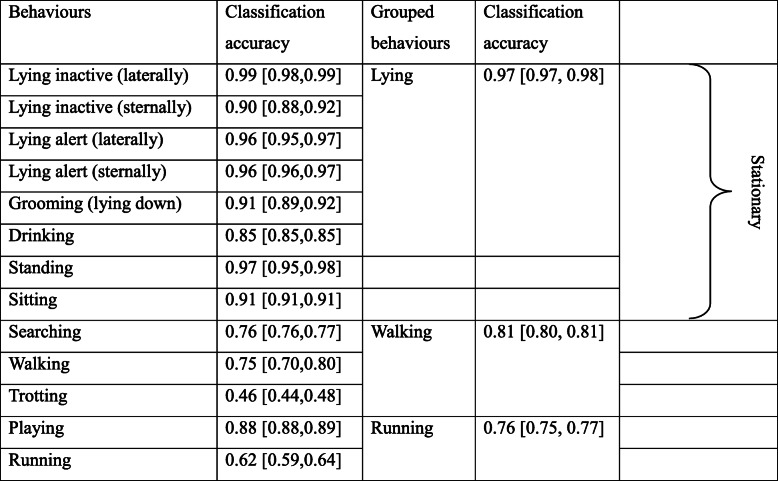
Performance of the Random Forest model at predicting 14 different behaviours versus grouped behaviours (from Tatler et al [30];). We combined similar behaviours to create three broad movement classes. The True Skill Statistic was used as our measure of classification accuracy, and the 95% confidence intervals are presented in square brackets next to each metric

The total acceleration recorded by accelerometers is the result of both static (gravitational) and dynamic (animal movement) components. Overall dynamic body acceleration uses the dynamic component and thus acceleration due to gravity must be removed. We calculated dynamic body acceleration (DBA) by subtracting a running mean (five seconds) from each acceleration axis (x, y, and z) to give acceleration values occurring from movement. The absolute value of DBA for each axis was then summed to give a per-second value of ODBA. A similar metric also derived from DBA, vectoral DBA (VeDBA), has also been shown to accurately predict energy expenditure for different behaviours in wild animals. Given Tatler et al. [30] found ODBA to be a better predictor of dingo behaviour than VeDBA, and that ODBA and VeDBA do not differ significantly in their ability to predict energy expenditure [19], we chose to estimate energy expenditure in dingoes using ODBA.

### Energy calculation

Time-energy budgets have been shown to be an effective estimate of daily energy expenditure when compared to doubly labelled water in previous trials (Weathers et al., 1984) and more recently ODBA was shown to accurately predict energy expenditure for specific activities [31]. We calculated daily energy expenditure using time-energy budgets calculated from our ACC derived behaviours and equations derived from the literature. For resting metabolic rate (applied to all stationary behaviours) we used oxygen consumption data from dingoes collected by Shield [32] and derived the following equation (Eq. 1) for V̇O_2_ against T_a_.

1 $$ \dot{V}{O}_2\ \left( ml\ {kg}^{-1}{\mathit{\min}}^{-1}\right)=0.007\times {T_a}^2-0.298\times {T}_a+9.968 $$

Where T_a_ was calculated per second using the Env-DATA system on Movebank (see ‘Environmental covariates’ section below). Shield [33] calculated *V̇*O_2_ using flow through respirometry from dingoes of a similar size to those in our study (mean ± se = 18.8 ± 0.2 kg vs 18.1 ± 0.4 kg) and respirometry was conducted across a similar temperature range to that experienced by the dingoes at Kalamurina. We selected *V̇*O_2_ data from the control group in Shield [33] as they were kept in an average ambient temperature of 23 °C over the course of their study, which was not distinctly different from the average ambient temperature at Kalamurina over our study period (26 ± 0.1 °C). For the purpose of this study, it was assumed that the rate of energy expenditure when sleeping is the same as when stationary as metabolic rate has not been measured in sleeping dingoes and we did not differentiate sleeping behaviour within our behavioural classifications. As such our calculations of daily energy expenditure may be slightly overestimated. For our walking and running behaviours we calculated energy expenditure using the following equation (Eq. 2) from Bryce and Williams [34] assuming an average speed of 1.985 m s^− 1^ for walking and 4.96 m s^− 1^ for running.

2 $$ \dot{V}{O}_2\ \left( ml\ {kg}^{-1}{\mathit{\min}}^{-1}\right)=7.5+6.16\times speed $$

We selected the ‘northern breed’ complex of domestic dogs (*Canis lupus familiaris*) as classified in Bryce and Williams [34] as these breeds most closely resemble dingoes in overall body size conditions and unfortunately, to the best of our knowledge, no data exist for *V̇*O_2_ of active dingoes. We did not account for ambient temperature during active behaviours because this is unlikely to have an additive effect on energy expenditure at low temperatures as heat generated from movement is often substituted for thermoregulation. Yet we cannot account for any additive effect ambient temperature may have on energy expenditure during activity in hyperthermic conditions. This is an entirely unstudied aspect of exercise physiology, with data only reported for a single individual primate [35]. Total daily energy expenditure was calculated per day for each individual by summing the cost of each activity multiplied by the time (in hours) each activity was undertaken. This was then converted to kJ kg^− 1^ day^− 1^ by multiplying by a factor of 20.1 [36].

### Environmental covariates

We created a map of the major landscape features in the study area using vegetation data from NatureMaps [37] and a spatial layer representing tracks and permanent water sources on Kalamurina provided by the Australian Wildlife Conservancy. We identified seven landscape features from the GIS data; watercourses, desert woodland, low shrubland, tracks, salt lakes, sand dunes, and flats. Landscape features provided varying amounts of shade based on vegetative cover, from full shade (desert woodland) to completely exposed (salt lakes). Each landscape layer was rasterized to the same resolution (25 m) and extent (56,366, 6,798,279; 339,166, 7,094,179; UTM Zone 54S) using the R package ‘raster’ [38]. Additional raster layers were generated from the landscape rasters by calculating the shortest distance from every cell to each landscape feature. Prior to statistical analysis, all such ‘distance to landscape feature’ variables were standardised (*x* – mean (*x*) / standard deviation (*x*)) and pairwise correlations (Pearson’s *r*) were calculated. Distance to flats was removed from the analyses because it was highly correlated with distance to sand dunes (*r* = 0.84). All other pairwise correlations were low (*r* < 0.7).

We used the Env-DATA system on Movebank to annotate environmental data (temperature, NDVI, rainfall, and wind speed) to each GPS location, with information sourced from the European Centre for Medium-Range Weather Forecasts [39] and NASA Land Processes Distributed Active Archive Center [40]. We collected data in two different field seasons, ‘winter’: April–August 2016, and ‘summer’ Oct 2017 – Jan 2018, and used the R library ‘Maptools’ [41] to calculate astronomical time of day (day, night, dawn, and dusk). We then grouped dawn and dusk together as ‘twilight’. We extracted hour and Julian day from our dataset as additional temporal covariates.

### Identification of high-use ‘shelters’

Dingoes repeatedly shelter in discrete areas for resting, rearing offspring, and/or socialising (hereafter referred to as ‘shelters’) and thus they may be an important predictor of energy use [42]. We used the R package ‘recurse’ [43] to identify shelters for each dingo by using a combination of 1) revisiting the same location (25 m radius), and 2) the average amount of time spent at that location (residence time). Shelters were defined individually for each dingo by an average residence time per visit of ≥60 min and a rate of recursion in the 90th percentile of all recursions, i.e., the highest rate of revisitation (Table 2).

**Table 2:** Attributes of the seven dingoes equipped with ACC-GPS collars at Kalamurina including the number of shelters, the mean (± se) daily distance travelled (km d-^1^), and daily energy expenditure (kJ kg-^1^ d-^1^)

ID	Sex	Weight (kg)	ACC collection period (days)	Total ACC fixes	Shelters	Daily energy expenditure	Distance travelled
JT04	F	16.0	12 Apr – 7 Aug 16 (44)	2,737,402	10	521 ± 1	6.9 ± 0.7
JT05	F	16.5	16 Apr – 3 Aug 16 (63)	2,652,399	3	611 ± 1	10.7 ± 0.6
JT07	M	20.5	12 Apr – 27 Apr 16 (4)	427,560	1	353 ± 1	3.9 ± 1.4
JT32	F	23.5	28 Oct – 11 Dec (25)	1,067,736	2	226 ± 3	11.2 ± 1.0
JT34	F	17.5	28 Oct – 24 Jan (46)	2,040,072	4	278 ± 4	9.1 ± 0.6
JT36	F	15.5	28 Oct – 24 Jan (64)	2,720,096	1	337 ± 4	12.8 ± 0.9
JT37	M	17.0	28 Oct – 24 Jan (46)	2,257,778	4	311 ± 4	15.5 ± 1.2

### Statistical analysis

#### Behaviour in space and time

Dingoes may exhibit different behavioural responses depending on their location in the landscape. So that individual differences in behaviour through space and time could be clearly identified, we chose to analyse the relationship between behaviour and landscape features for each dingo separately, using a multinomial logistic regression in the R package ‘MDM’ [44]. Our dependent variable was the proportion of time a dingo was engaged in each behaviour (stationary, walking, and running) in the 900 s (i.e., 15 min) prior to the GPS fix, with landscape feature as our predictor variable. To investigate population-level seasonal differences in dingo behaviour, we performed a meta-analysis to generate global parameter estimates across dingoes tracked during winter (JT04, JT05, and JT07) and summer (JT32, JT34, JT36, and JT37). We weighted the estimates from the individual models by the inverse of each estimate’s standard error, to account for variation in the sample size.

#### Daily activity

To investigate daily activity patterns of dingoes at Kalamurina we constructed two generalised additive models (GAMs) using the R package ‘mgcv’ [45]. Our first activity model assessed movement distance (between successive 15-min GPS locations) as a function of hour of day (0–23). Similarly, the second activity model assessed ODBA (averaged across the same, preceding 15-min period as the distance measure) as a function of hour. Prior to statistical analysis, both response variables were standardised (*x* – mean (*x*) / standard deviation (*x*)). GAMs were fitted with a cyclic cubic regression spline and 20 knots. We also ran a Pearson’s correlation to test the strength and direction of the correlation between the response variables.

To assess whether dingoes exhibited behavioural thermoregulation by adjusting their activity levels as a result of ambient temperature, we used a generalised linear model (GLM) with ODBA (standardised) as our response variable, and ambient temperature (standardised) and time of day (day or night) as our predictors. We included dingo ‘ID’ and ‘Julian day’ as random effects.

#### Effect of landscape features on behaviour

We used linear mixed effect models in the R package ‘lme4’ [46] to explore the relationship between (log transformed) ODBA values (using 5 s average around each GPS timestamp) and our environmental/temporal covariates. Based on our aims and a priori assumptions of dingo activity, we built a candidate set of models (*n* = 25) and used Akaike’s Information Criterion corrected for small sample sizes (AICc) and conditional R^2^ (R package ‘MuMin’ [47];) to rank the models. All candidate models included dingo ID as a random effect to account for individual variation.

## Results

**Fig. 2:**
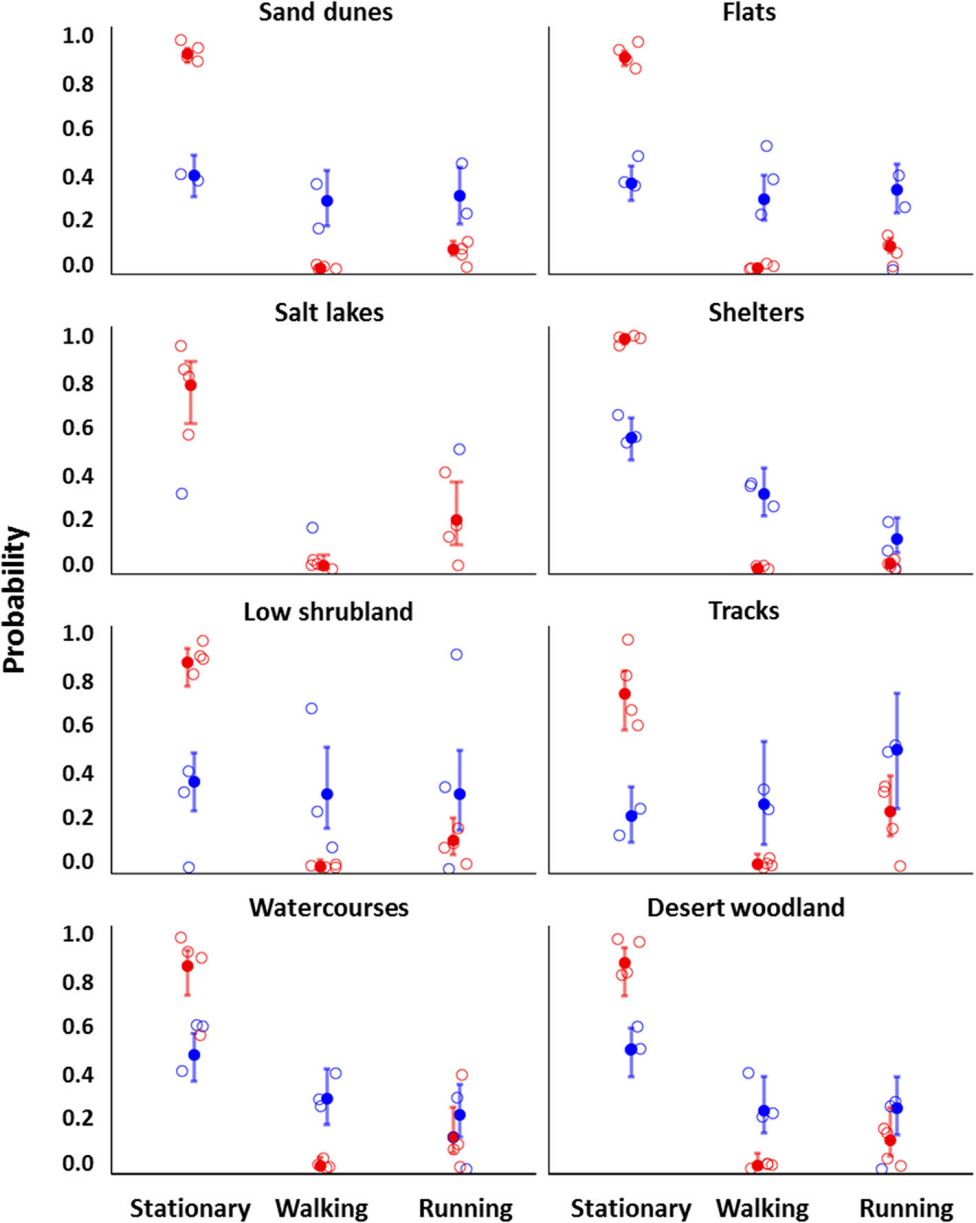
Dingoes were much less active during summer than they were in winter. Predicted probabilities of being stationary, walking, or running in each habitat. Blue represents dingoes tracked during winter (*n* = 3), and red represents dingoes tracked during summer (*n* = 4). Hollow circles indicate probabilities for individual dingoes and solid circles represent global estimates (± 95% confidence intervals) from our meta-analysis of the multinomial model estimates for each individual. In winter, only one dingo occurred on salt lakes, so no global statistic was calculated

Dingoes were much less active during summer where 91 ± 0.04% (mean ± sd) of their day (24 h) was spent stationary versus only 46 ± 0.1% during winter (Table S1). Season had the most profound effect on dingo behaviour (Fig. 2). In summer, dingoes were much more likely to remain stationary than any other behaviour, regardless of where they were in the landscape. In contrast, dingoes were just as likely to be stationary as they were to be walking or running during winter. The model with the lowest AICc and highest R^2^ (0.57) nested behaviour within ID, included Julian day as a random effect, and landscape feature, time of day, and the interaction between time of day and season as fixed effects.

**Fig. 3:**
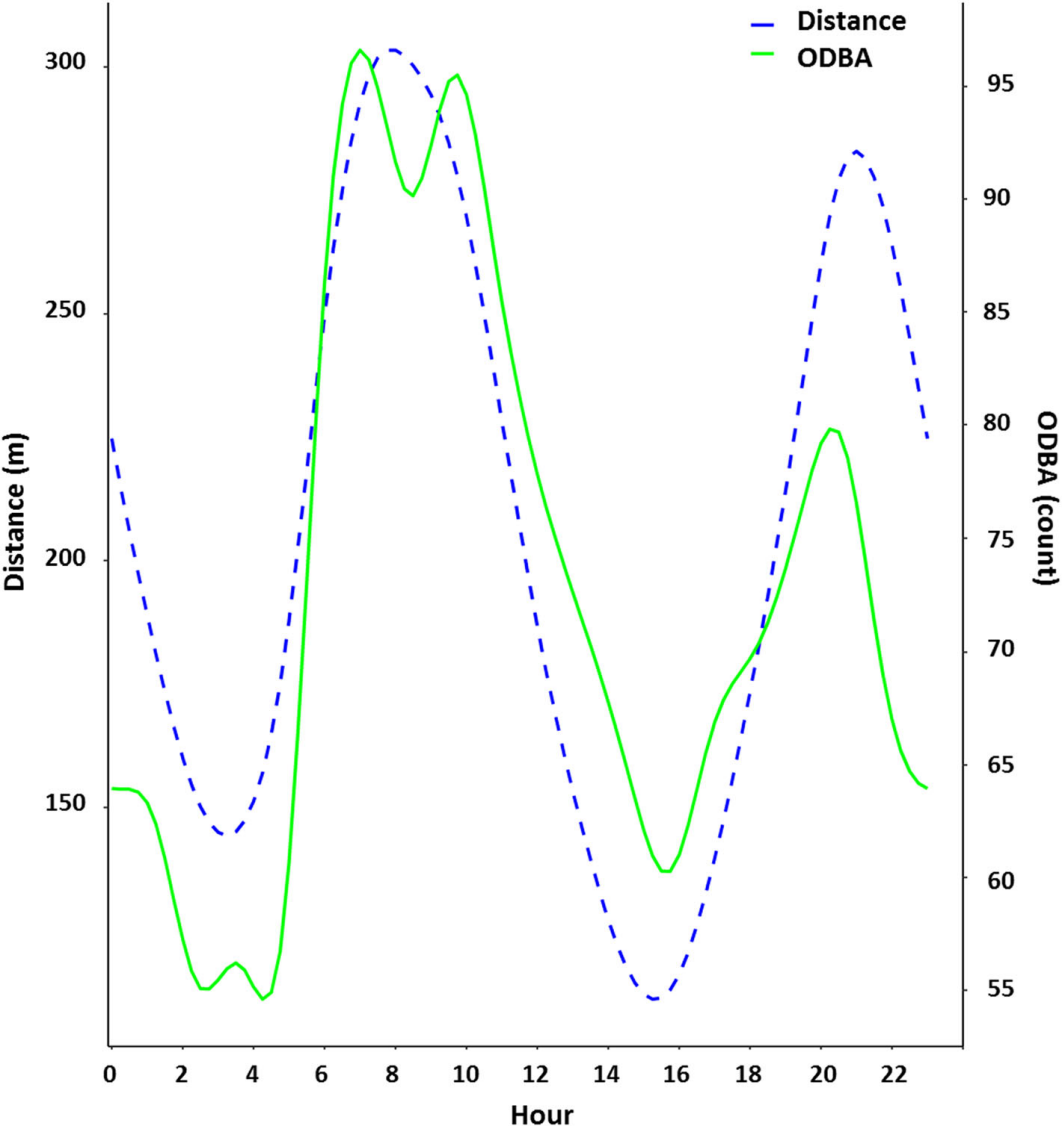
Distance and ODBA were positively correlated with each other (*r* = 0.65, *p* < 0.001), and both of these variables indicated crepuscular patterns of activity. Daily activity patterns of dingoes (*n* = 7) at Kalamurina. The blue dotted line represents the distance moved between successive 15 min GPS location, and the solid green line represents the predicted, mean ODBA value across 900 s (i.e., 15 min), as a function of hour of day. The axis for ODBA has been scaled to fit within the range of the predicted distance values

Distance and ODBA were positively correlated with each other (*r* = 0.65, *p* < 0.001), and both variables indicated crepuscular patterns of activity (Fig. 3). Dingoes were most active at dawn and into the early hours of the night and least active just before dawn and in the middle of the afternoon when compared across the entire study period. However, during winter dingoes were significantly less active during twilight but more active at night than dingoes in summer. The overall activity level of dingoes during the day was not significantly different between summer and winter.

**Fig. 4:**
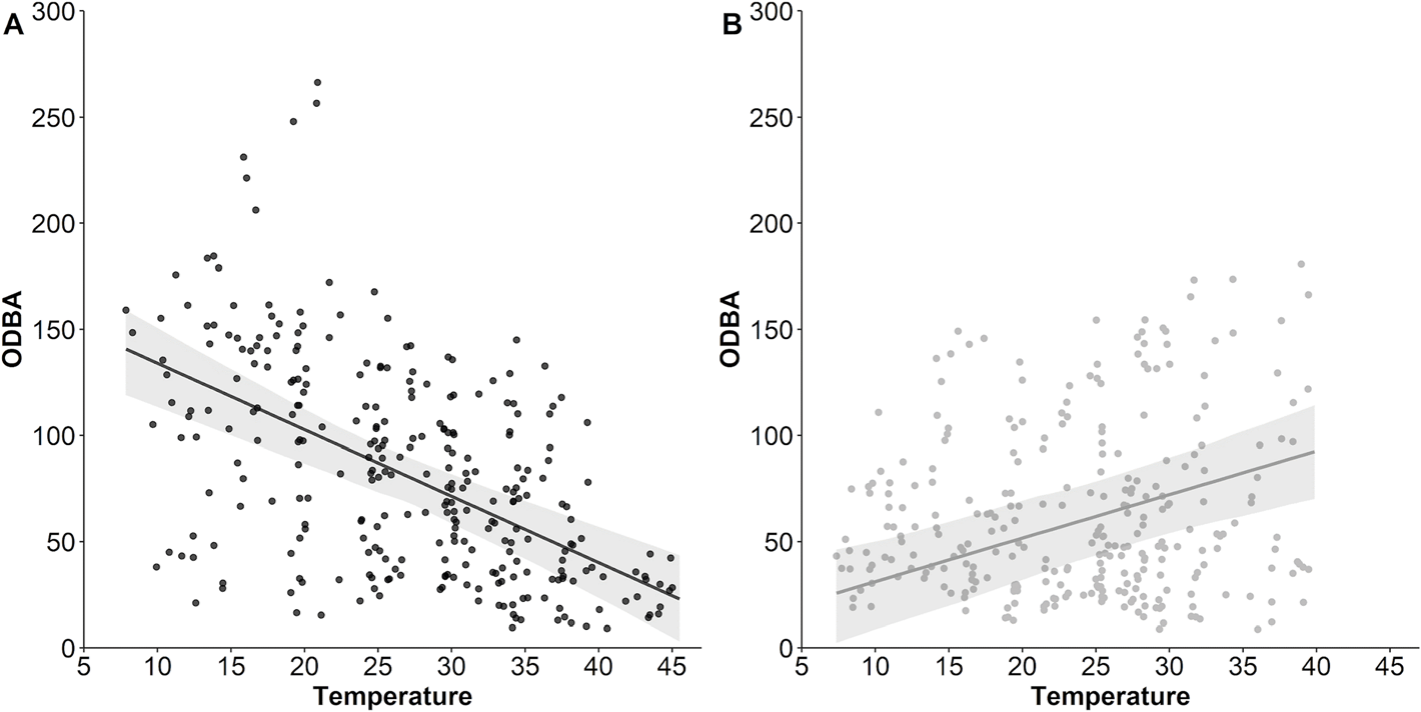
Predicted ODBA values (activity) by ambient temperature for dingoes (*n* = 7) at Kalamurina either during the day (Panel **a**) or at night (Panel **b**). The 95% confidence intervals are represented by grey shading

We found a contrasting relationship between ODBA and T_a_ that was driven by the time of day (i.e. whether it was day or night; Fig. 4). There was a sharp decrease in ODBA with increasing T_a_ during the day (*R*^2^ = 0.59), whereas ODBA increased with increasing T_a_ at night (*R*^2^ = 0.39). Estimates of mean daily energy expenditure are shown in Table 2. The mean estimated energy expenditure of dingoes was significantly higher in winter (495 kJ kg^− 1^ day^− 1^) than summer (288 kJ kg^− 1^ day^− 1^; *p* = 0.03).

**Fig. 5:**
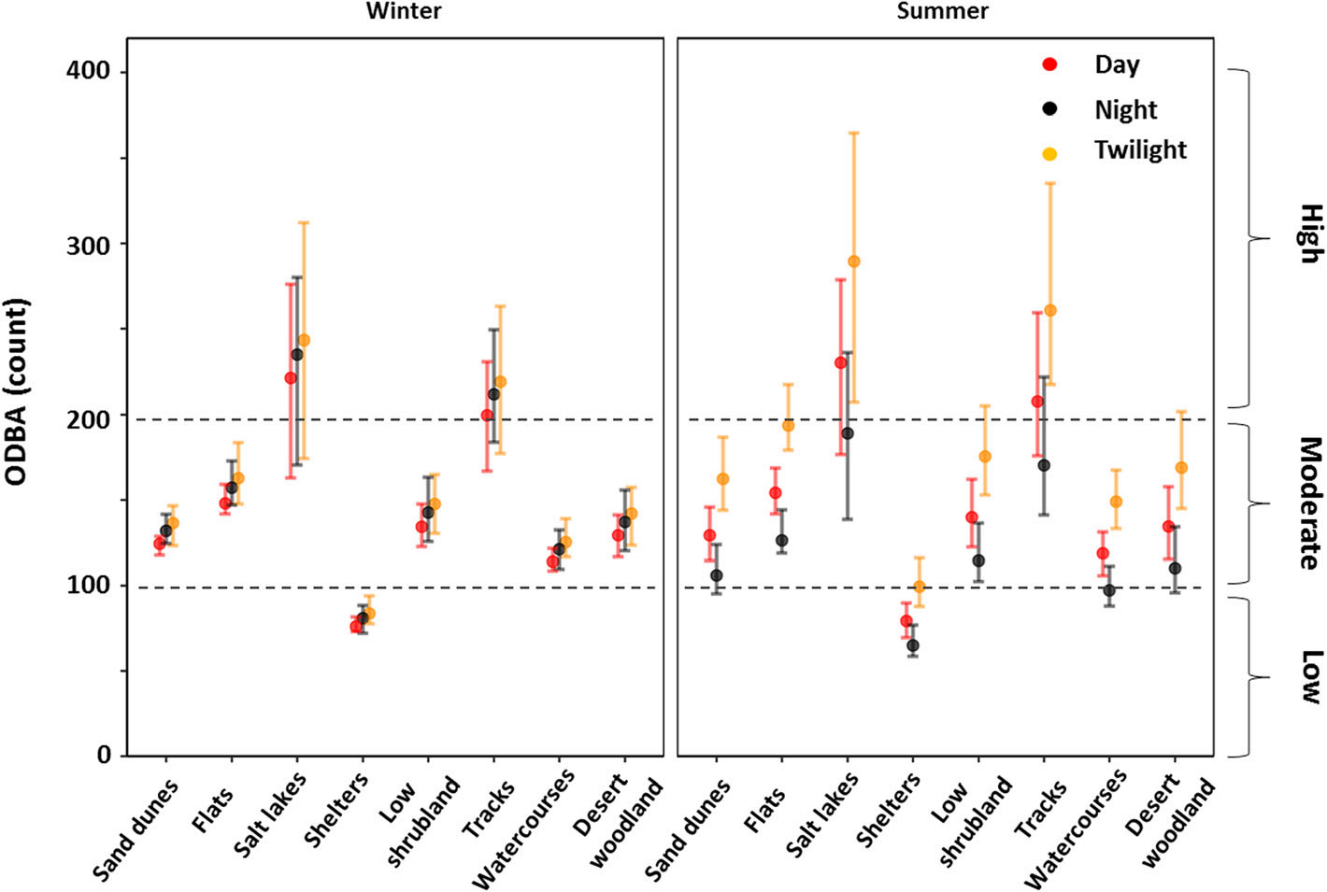
Predicted ODBA values from our selected generalised linear mixed-effect model for dingoes (*n* = 7) in eight landscape features during the day, night, and twilight. Approximate activity levels (low, moderate, and high) were adapted from the relationship between ODBA and behaviour reported in Tatler et al. [30] and broadly represent our three grouped behaviour classes (stationary, walking, and running), respectively. Error bars represent 95% confidence intervals

There was a significant effect of landscape feature on the activity levels (ODBA) of dingoes at Kalamurina (Fig. 5). Dingoes were most active on salt lakes, tracks, and flats, and least active when at their shelters (Table S2). However, the time of day had a significant effect on how active dingoes were in each landscape feature during summer but not in winter (Fig. 5). Overall, dingoes exhibited a moderate - low level of activity in most landscape features.

## Discussion

Patterns and processes of all life in the arid zone are shaped by extremes in temperature and water availability. Kalamurina is one of the hottest and driest places in Australia with a long term median annual rainfall < 135 mm and maximum temperatures regularly above 40 °C throughout summer. This region is predicted to experience an increase in heat related extremes and duration of warm spells which could triple the number of days above 40 °C by 2090 [24]. In our study the activity of arid zone dingoes, as measured by ODBA, was primarily driven by ambient temperature. More specifically, we found that this was reflected in activity patterns across time of day, season, and even landscape features. Our results suggest that under future climate scenarios dingoes may shift their behaviour to avoid hyperthermia.

Dingoes in this study were largely crepuscular, with two troughs in activity occurring during the early hours of the morning and the hottest part of the day (mid-afternoon). Akin to other animals, the movement ecology of dingoes is influenced by seasonally-variable intrinsic and extrinsic factors, with either primarily diurnal or primarily nocturnal activity patterns reported in other studies [42, 48]. Activity patterns of predators usually coincide with those of their major food source, which are also linked to ambient temperature [49, 50]. Rabbits comprise the bulk of the dingo’s diet in the arid zone [27], with most rabbit activity occurring at night regardless of season [51]. The time of day and seasonal differences in the activity of dingoes in this study is therefore unlikely to be driven solely by prey acquisition. Moreover, as a vagile species, constraining high activity movements (or reducing them altogether) to the less climatically extreme times of the day is likely a behavioural adaptation to mitigate thermal stressors associated with desert life [5, 52, 53]. While we were unable to track the activity of the same individuals across seasons, different patterns of daily movement during summer and winter have been shown to occur within individual dingoes in this study system [42]. This suggests that our findings are more likely a response to temperature variation between seasons and not simply an artifact of differences between individuals.

Seasonally driven activity constraints have been reported for other species (e.g., flying squirrels [54]*,* and desert woodrats [55]) and suggests a trade-off between remaining in areas which offer thermal respite versus obtaining resources. Seasonal differences in daily energy expenditure in free-ranging eutherian mammals have been found in several species, though this is the first time it has been observed in a wild canid. It has been shown that dingoes are capable of acclimating physiologically to extreme temperatures (− 41 °C to + 45 °C) over the course of a few months by reducing their metabolic rate [33]. Warm acclimated individuals exposed to temperatures up to 45 °C reduced their metabolic rate by around 40% compared to control individuals. This was observed in concert with a change in thermal conductance, an important component of heat dissipation, brought about by altered coat composition [33]. It is reasonable to assume that these physiological changes could contribute to seasonal changes in daily energy expenditure for dingoes in our study, however behavioural thermoregulation via altered activity patterns remains an important facet of energy balancing exhibited by dingoes.

Movement is energetically costly and evaporative water loss is highest during energetically demanding activities at high ambient temperatures [1, 56]. During locomotion even at low ambient temperatures (< 10 °C) canines can rapidly reach high body temperatures (15–20 minutes to reach 42 °C), due to the heat produced by muscle activity [57]. Therefore, at high ambient temperatures the additive heat produced during activity must be actively dumped, potentially further increasing energetic costs. As such dingoes would benefit from remaining inactive in the heat in order to reduce hyperthermia and evaporative water loss. We found that dingoes were stationary for approximately 22 h a day during summer compared to only 12 h during winter. Winter also coincided with the breeding and whelping seasons, which could also explain why dingoes were more active during this time (e.g., searching for mates). Further, the daily energy expenditure of the two female dingoes tracked in winter was considerably higher than the male’s, which may be a consequence of increased metabolic demands associated with lactation. Activity levels of lactating females rise in response to increased foraging effort due to additional energetic demands and fluid requirements for milk production, which can be twice those of basal needs [58]. However, as we were unable to make direct observations of reproductive status this is merely speculative.

Dingoes displayed the highest activity levels on salt lakes and tracks, which was expected given they are primarily used for commuting [42]. These exposed parts of the landscape are likely used as directional travel routes between resources such as water, shelter, and food but are also important for communication as dingoes mark their territory by depositing visual and olfactory cues (e.g., faeces and urine) in conspicuous places to maximise their detection by conspecifics. Returning to discrete areas for shelter and/or denning is common amongst mammalian carnivores and can increase individual fitness by providing thermoregulatory benefits [59], reducing predation rates [60], and increasing offspring survival rates [61]. Further, microclimate selection (i.e., location of shelters in the landscape) is an important thermal defence employed by animals to buffer changes in ambient temperature. We previously collected data on the same dingo population and found that shelters were significantly more likely to be located in the densely vegetated desert woodlands and along watercourses than in exposed habitats like salt lakes [42].

Regardless of season, we found evidence that dingoes behaviourally thermoregulate by decreasing their activity levels with increasing ambient temperature during the day. Conversely, the positive relationship between activity and temperature at night suggests that dingoes could be compensating for low daily activity by partially shifting their movement to nocturnal periods to avoid solar radiation. As the most energetically costly behaviours for dingoes occur in exposed landscapes, shifting movements to the night would mitigate the issue of radiative heat gain while still enabling dingoes to perform important behaviours, such as hunting and socialising.

## Conclusions

Understanding the flexibility of behavioural thermoregulation in the arid zone informs our understanding of how populations or species will respond to a changing climate. Here, we suggest that the behavioural ecology of a medium sized carnivore in the arid zone is driven by limitations to heat dissipation regardless of season, in line with the hypothesis that heat dissipation limits the upper boundary of total energy expenditure [22]. The shifts in behaviour observed in this study in response to increasing ambient temperatures have also been reported for other arid zone carnivores, such as Namibian cheetahs (*Acinonyx jubatus*) and African wild-dogs (*Lycaon pictus*) [62, 63]. Given the previously reported physiological capacity of dingoes to acclimate to temperatures exceeding 40 °C within months [33], alongside evidence of behavioural thermoregulation reported here, it appears that dingoes may be equipped to survive a predicted increase in temperatures in their environment, albeit via behavioural shifts. This may reflect how other apex predators in arid environments will respond to climate change and could have significant repercussions for predator-prey dynamics and intraguild competition in these ecosystems.

## Supplementary Information

**Additional file 1: Table S1.** Proportion of each day spent stationary, walking, and running. **Table S2.** Model summary showing the effect of landscape features, time of day, and period on dingo activity (ODBA). Model estimates, standard errors (SE) and *p*-values for our correlated intercepts and slopes linear mixed model are presented. Significance is indicated in bold

## Data Availability

Data found in this manuscript is made available via Figshare: 10.25909/5c901de204d18
