# Supplementary material for: Accelerometer informed time-energy budgets reveal the importance of temperature to the activity of a wild, arid zone canid
Source: Mov Ecol. 2021 Mar 18;9:11. doi: 10.1186/s40462-021-00246-w (PMC7977315; doi:10.1186/s40462-021-00246-w)
Supplement: Supplementary file 1 — Additional file 1: Table S1. Proportion of each day spent stationary, walking, and running. Table S2. Model summary showing the effect of landscape features, time of day, and period on dingo activity (ODBA). Model estimates, standard errors (SE) and p-values for our correlated intercepts and slopes linear mixed model are presented. Significance is indicated in bold [file 40462_2021_246_MOESM1_ESM.docx]

Table S1. Proportion of each day spent stationary, walking, and running.

| Dingo ID | Sex | Stationary | Walking | Running | Season |
| --- | --- | --- | --- | --- | --- |
| JT04 | F | 0.47 | 0.35 | 0.18 | autumn-winter |
| JT05 | F | 0.38 | 0.23 | 0.40 | autumn-winter |
| JT07 | M | 0.54 | 0.46 | <0.01 | autumn-winter |
| JT32 | F | 0.97 | 0.02 | 0.01 | spring-summer |
| JT34 | F | 0.93 | 0.01 | 0.07 | spring-summer |
| JT36 | F | 0.87 | 0.01 | 0.12 | spring-summer |
| JT37 | M | 0.88 | 0.02 | 0.10 | spring-summer |

Table S2. Model summary showing the effect of landscape features, time of day, and period on dingo activity (ODBA). Model estimates, standard errors (SE) and p-values for our correlated intercepts and slopes linear mixed model are presented. Significance is indicated in bold.

| Model | Variables | Estimate | SE | *p* value |
| --- | --- | --- | --- | --- |
| log(ODBA) ~ (1 + Behaviour\|ID) + (1\|J.day) +  Landscape feature +  Time of day +  Period:Time of day | **(Intercept)** | **4.818** | **0.055** | **< 0.001** |
|  | **Flats** | **0.175** | **0.029** | **< 0.001** |
|  | **Salt lakes** | **0.593** | **0.120** | **< 0.001** |
|  | **Shelter** | **-0.495** | **0.040** | **< 0.001** |
|  | Low shrubland | 0.055 | 0.071 | 0.439 |
|  | **Tracks** | **0.486** | **0.083** | **< 0.001** |
|  | Watercourses | -0.075 | 0.054 | 0.166 |
|  | Desert woodland | 0.026 | 0.065 | 0.688 |
|  | **Night** | **-0.174** | **0.035** | **< 0.001** |
|  | **Twilight** | **0.235** | **0.036** | **< 0.001** |
|  | Day:Winter | 0.000 | 0.071 | 0.998 |
|  | Night:Winter | 0.217 | 0.072 | 0.005 |
|  | Twilight:Winter | -0.174 | 0.074 | 0.022 |
